# Composition dependence of charge and magnetic length scales in mixed valence manganite thin films

**DOI:** 10.1038/srep29632

**Published:** 2016-07-27

**Authors:** Surendra Singh, J. W. Freeland, M. R. Fitzsimmons, H. Jeen, A. Biswas

**Affiliations:** 1 Solid State Physics Division, Bhabha Atomic Research Centre, Mumbai 400085 India; 2 Advanced Photon Source, Argonne National Laboratory, Argonne, Illinois 60439, USA; 3 Quantum Condensed Matter Division, Oak Ridge National Laboratory, Oak Ridge, TN, USA; 4 Department of Physics, University of Florida, Gainesville, FL 32611, USA; 5 Department of Physics, Pusan National University, Busan 609-735, Korea

## Abstract

Mixed-valence manganese oxides present striking properties like the colossal magnetoresistance, metal-insulator transition (MIT) that may result from coexistence of ferromagnetic, metallic and insulating phases. Percolation of such phase coexistence in the vicinity of MIT leads to first-order transition in these manganites. However the length scales over which the electronic and magnetic phases are separated across MIT which appears compelling for bulk systems has been elusive in (La_1−y_Pr_y_)_1−x_Ca_x_MnO_3_ films. Here we show the in-plane length scale over which charge and magnetism are correlated in (La_0.4_Pr_0.6_)_1−x_Ca_x_MnO_3_ films with x = 0.33 and 0.375, across the MIT temperature. We combine electrical transport (resistance) measurements, x-ray absorption spectroscopy (XAS), x-ray magnetic circular dichroism (XMCD), and specular/off-specular x-ray resonant magnetic scattering (XRMS) measurements as a function of temperature to elucidate relationships between electronic, magnetic and morphological structure of the thin films. Using off-specular XRMS we obtained the charge-charge and charge-magnetic correlation length of these LPCMO films across the MIT. We observed different charge-magnetic correlation length for two films which increases below the MIT. The different correlation length shown by two films may be responsible for different macroscopic (transport and magnetic) properties.

Colossal magnetoresistance (CMR)^1^ perovskite manganites exhibit numerous phase transitions^2^,^3^,^4^,^5^,^6^,^7^,^8^,^9^, like metal–insulator transition (MIT), ferromagnetic–paramagnetic (FM–PM) and structural phase transitions. A rich variety of electronic and magnetic phases often coexist and compete with one another^1^,^2^,^3^,^4^,^5^,^6^,^7^,^8^,^9^ in mixed phase manganites that can be used to achieve interesting functionalities. It is well recognized that many mixed-valence manganites phase separate between the FM metallic phase and the charge-ordered (CO) insulating phases. When driven by disorder near first-order transitions, the length scale of phase-separated domains can be much less than a micron^2^,^9^,^10^,^11^,^12^.

Bulk (La_1−y_Pr_y_)_1−x_Ca_x_MnO_3_, is a system^6^,^11^,^12^,^13^,^14^ exhibiting phase separation in the range of nanometers to microns for x = 0.33^9^ and 0.375^11^. Recently Moshnyaga *et al.*^15^ predicted that electronic phase separation (EPS) in thin films of a (La_1−y_Pr_y_)_1−x_Ca_x_MnO_3_ with x = 0.33 and y = 0.40, develops at the nanometer scale, in which adjacent FM nanodomains are antiferromagnetic (AFM) coupled. The EPS at nanometer length scale at the surface of (La_0.4_Pr_0.6_)_1−x_Ca_x_MnO_3_, (LPCMO) film with x = 0.33 was observed using conductive atomic force microscope (cAFM)^16^. Further a combination of cAFM^16^ and transport measurements as a function of applied stress^17^ on LPCMO film with x = 0.33 also suggested that the growth of anisotropic electronic (metallic) domain resulting into anisotropic response of the MIT and resistance of the sample.

Recently using polarized neutron reflectivity we observed that magnetic ordering in LPCMO film with x = 0.33 was not caused by the metal-insulator transition; rather magnetic ordering first occurs at higher temperatures^18^. In addition the results also indicated a magnetic percolation threshold of 0.6 (assuming metallic and ferromagnetic phases are from same parts of the LPCMO film), which is consistent with two-dimensional spin lattices. However, direct evidence for coexistence of magnetic and nonmagnetic regions and their length scales has been elusive in LPCMO films.

Here we show that the in-plane length scales over which charge and magnetism are correlated in (La_0.4_Pr_0.6_)_1−x_Ca_x_MnO_3_ films with x = 0.33 and 0.375 are different. Two single crystal (La_0.4_Pr_0.6_)_1−x_Ca_x_MnO_3_ (LPCMO) films with x = 0.33 and 0.375, hence forth known as samples S1 and S2, respectively, were epitaxially grown on (110) NdGaO_3_ (NGO) substrates^19^,^20^,^21^. Using x-ray absorption spectroscopy (XAS), x-ray magnetic circular dichroism (XMCD), and specular/off-specular x-ray resonant magnetic scattering (XRMS)^22^,^23^,^24^ we quantitatively estimated charge-charge and charge-magnetic correlation length of the samples across MIT. We observed smaller in-plane charge correlation length for LPCMO film with x = 0.33 than that of film with x = 0.375, which is consistent with the length scale associated with conductivity map of the films. We also observed different charge-magnetic correlation length for two films which increases below the MIT. However both chemical and magnetic surfaces show similar fractal dimension of ~2.7 with similar atomic scale roughness ~4 Å.

## Results

### Transport and conductivity measurements

**Figure 1:**
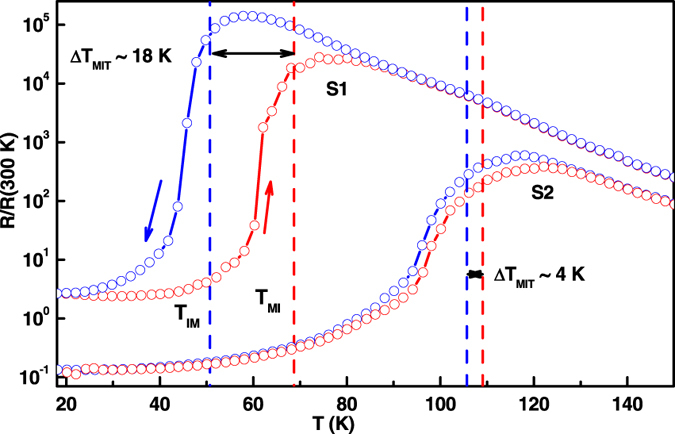
Electrical transport (resistance, R) measurements from samples S1 and S2.

The electrical transport (resistance) measurements were taken using the two-probe method^25^ in a closed cycle helium cryostat. The temperature was varied between room temperature to 20 K with an accuracy of better than 0.1 K. Figure 1 shows resistance normalized to the 300 K resistance [*R*(*T*)/*R*(300 K)] from S1 and S2. We cooled and warmed our samples at a rate of 0.4 K/min. We obtained an insulator to metal (while cooling) (*T*_IM_) and metal to insulator (while warming) (*T*_MI_) transition temperatures (location of the peaks of d*R*/d*T*) of 50.7 K and 68.7 K, respectively—a difference of Δ*T* ~18 K for S1. Locations of *T*_IM_ and *T*_MI_ are shown as dashed lines in Fig. 1. However, the MIT for S2 occurred at higher temperatures (*T*_IM_ = 105.6 K and *T*_MI_ = 109.0 K) with a smaller thermal hysteresis ~4 K. We have grown number of samples S1 and S2 and observed similar variation of *R*(T) [See Supplementary Fig. S1] as shown in Fig. 1. For similar thickness of samples S1 and S2, we observed that sample S2 shows MIT at higher temperature, which is ~40 K more than that of sample S1, suggesting a composition dependent variation of macroscopic properties (resistance) with respect to temperature. Earlier macroscopic magnetization measurements on similarly grown sample S1 have suggested a Curie temperature of ~130 K^19^,^20^,^21^. The Curie temperature for similarly grown S2 is ~150 K [See Supplementary Fig. S2].

**Figure 2:**
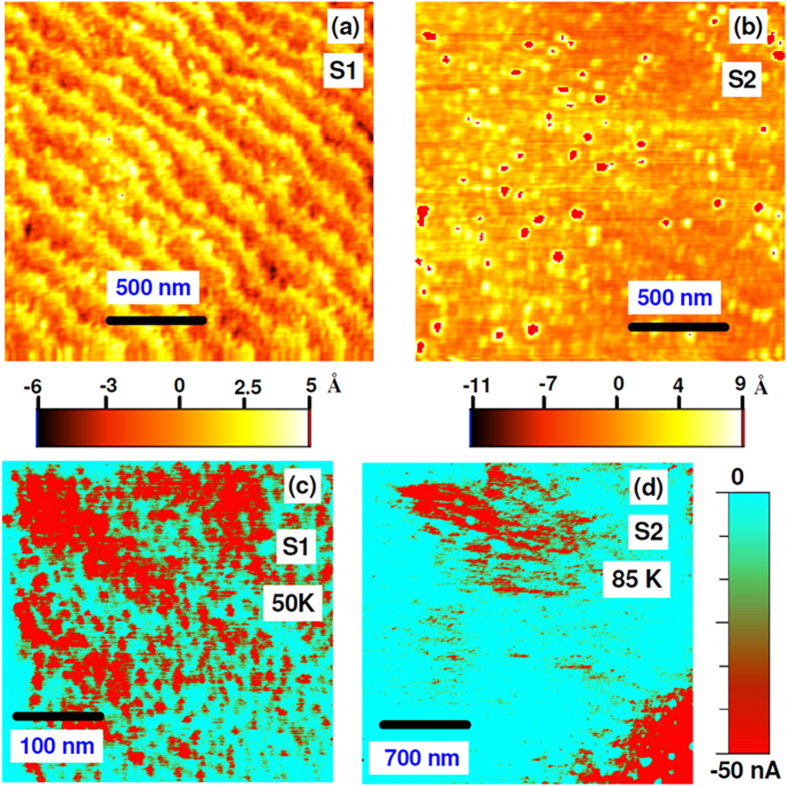
Topography image with a scan size of 2 μm × 2 μm, of the surface of LPCMO samples S1 (**a**) and S2 (**b**). (**c**,**d**) show the current distribution measured by conducting atomic force microscopy for S1 (scan area: 0.4 μm × 0.4 μm) and S2 (scan area: 2.8 μm × 2.8 μm), respectively.

Temperature dependent morphology and conductance measurements on the surface of similarly grown LPCMO film with x = 0.33 (S1) were reported elsewhere^16^. For cAFM measurements both the samples were cooled to lowest temperature of ~45 K and then measurements were taken during the cooling and warming cycle at different temperatures. The *R*(*T*) measurements for the samples on which cAFM measurements were made are different [See Supplementary Fig. S3]. Figure 2(a,b) depict the topography with a scan area of 2 μm × 2 μm of identically grown samples S1 and S2, showing distinct morphologies of the two samples. However the atomic scale morphological parameters, roughness and roughness exponent, for two films as obtained from off-specular x-ray reflectivity (XRR), discussed latter, are similar. To compare the conductivity map we have measured the cAFM images of the surfaces of identically grown samples S1 and S2 at a temperature below the *T*_MI,_ while warming the samples. Figure 2(c,d) show the cAFM images at 50 K and 85 K with scan areas of 0.4 μm × 0.4 μm and 2.8 μm × 2.8 μm of the surface of identically grown samples S1 and S2, respectively. The cAFM images show the existence of metallic and insulating phases. The maximum size of metallic domains [Fig. 2(c)] at 50 K for S1 was ~1700 Å, whereas for S2 it was ~9000 Å at 85 K [Fig. 2(d)]. We observed even larger metallic domains (~12000 Å) for S2 at 50 K (not shown here).

### Hard (non-resonant) X-ray reflectivity

**Figure 3:**
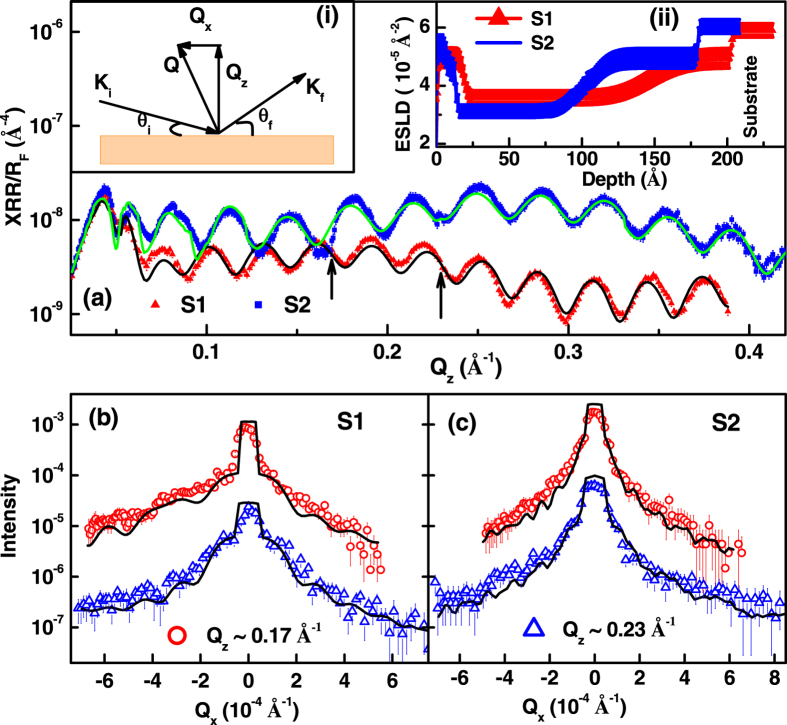
(**a**) Specular hard X-ray reflectivity (XRR) data from samples S1 and S2. Arrow in (**a**) indicate the value of Q_z_ where we have performed off-specular XRR. Inset (i) and (ii) of (**a**) show the scattering geometry and electron scattering length density (ESLD) depth profiles for S1 and S2, which best fitted (solid lines in (**a**)) the XRR data. (**b**,**c**) show the off-specular XRR from S1 and S2, respectively. Off-specular XRR in (**b**,**c**) for two Q_z_ are shifted by a factor of 10 for better visualizations.

XRR involves measurement of the x-ray radiation reflected from a sample as a function of wave vector transfer *Q* (i.e., the difference between the outgoing and incoming wave vectors) [inset (i) of Fig. 3]. In case of specular reflectivity (angle of incidence, *θ*_i_ = angle of reflection, *θ*_f_), 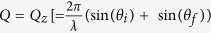, where λ is the x-ray wavelength] and 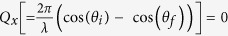, thus we obtain the depth dependent chemical profile of the sample^26^. Whereas in the case of off-specular reflectivity (*θ*_i_ ≠ *θ*_f_), the reflectivity (as a function of *Q*_x_ at fixed *Q*_z_) provides information about the correlation of structure across the sample plane^26^,^27^,^28^,^29^,^30^, e.g. roughness. The x-ray specular reflectivity is qualitatively related to the Fourier transform of the electron scattering length density (ESLD) depth profile*ρ*(*z*) averaged over the whole sample area^26^,^27^.

The specular reflectivities were calculated using the dynamical formalism of Parratt^31^, and parameters of the model were adjusted to minimize the value of reduced *χ*^2^ –a weighted measure of goodness of fit^32^. The specular XRR data, normalized to the asymptotic value of the Fresnel reflectivity^26^ 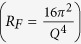, from S1 (closed triangle) and S2 (closed square) are shown in Fig. 3(a) along with the best fit (solid lines). Inset (ii) represents the ESLD depth profile of these samples which best fitted the specular XRR. We obtained a thickness of 200 ± 10 and 180 ± 10 Å for LPCMO layer in S1 and S2, respectively, from specular XRR^33^. Different ESLD model with different *χ*^2^ for fitting specular XRR data is reported in ref. 33. We obtained chemical non uniformity at both the interfaces (air-film and film-substrate) resulting to different ESLD profiles at interfaces for both films^20^,^21^,^33^.

Investigation of interface morphology of thin film, using the distorted-wave Born approximation (DWBA) formalism, was developed by Sinha *et al.*^29^ which has been subsequently extended to multilayer hetrostructures^34^,^35^,^36^,^37^. We collected off-specular (diffuse) reflectivity at different angles of incidence in the scattering plane termed as detector scan^35^. Figure 3(b,c) show the off-specular XRR data as a function of *Q*_x_ at two values of *Q*_z_, (= 0.17 Å^−1^ and 0.23 Å^−1^) from S1 and S2, respectively. We analyzed the off-specular XRR measurements from the samples under the approximation of self-affine fractal surface, where in-plane height-height correlation function *C*(*x*, *y*) is usually assumed^29^,^34^,^35^,^36^,^37^: 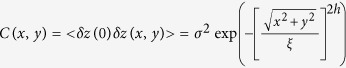; where σ is the *rms* value of the surface roughness (correlated roughness), *h* is the roughness exponent, known as Hurst parameter and *ξ* is the in-plane correlation length of the roughness. The exponent 0 < *h* < 1 determines the fractal dimension (*D* = 3–*h*) of the interface (i.e., how jagged the interface is; smoother interfaces have larger values of *h*)^29^. The off-specular XRR were simulated using the formalism developed by Holý *et al.*^34^ to obtain the incoherent diffuse scattering cross-section (see Eq. (16) in ref. 34). The σ, *h* and *ξ* for each interface are the parameters of the fit to off-specular XRR data while other parameters obtained from the specular XRR (i.e. thickness and electron density) were kept fixed. We fitted off-specular reflectivity as a function of *Q*_x_ at two values of *Q*_z,_ with the same set of parameters (Table 1). Fits to the off-specular XRR are shown as solid line in Fig. 3(b,c). Errors reported for parameters obtained from both hard and soft (discussed later) x-ray scattering measurements represent the perturbation of a parameter that increased χ^2^ corresponds to a 2σ error (95% confidence)^32^. It is evident from the analysis of off-specular hard XRR (Table 1) that samples show drastically different charge-charge (height-height) correlation length even though they show similar atomic scale roughness (*σ*) and roughness exponent (*h*), this may be due to different composition (doping) of the films. However the contribution of height-height distribution (surface morphology) from two films which are drastically different (AFM images) can lead to different correlation length.

### X-ray absorption spectroscopy and X-ray resonant scattering

The XAS and XMCD were calculated by averaging and taking the difference of the photocurrent signals from each polarization (right (*I*^+^) and left (*I*^−^) circularly polarized), (*I*^+^ + *I*^−^)/2 and (*I*^+^ − *I*^−^), respectively. The XMCD is proportional to an average near-surface magnetic moment of a few nanometers^23^,^24^. Since XRMS relies on measuring the reflected x-ray beam, it is sensitive to the magnetization depth profile of a few tens of nanometers^23^,^24^.

**Figure 4:**
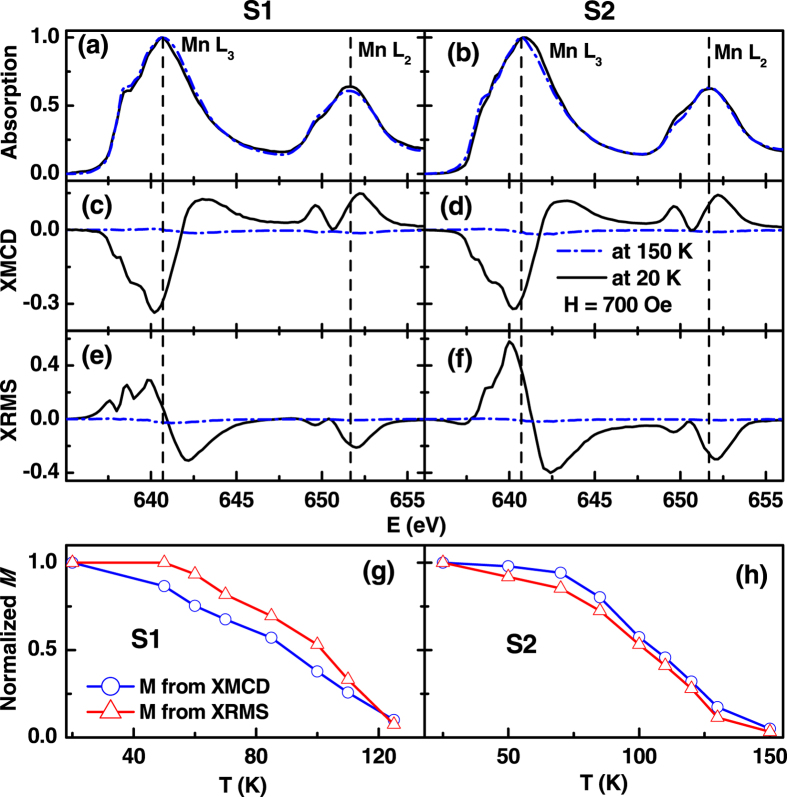
Near surface x-ray absorption from samples S1 (**a**) and S2 (**b**) at 150 K (dash curves) and 20 K (solid curves). XMCD spectra from samples S1 (**c**) and S2 (**d**) at 150 K (dash curves) and 20 K (solid curves). XRMS spectra from samples S1 (**e**) and S2 (**f**) at 150 K (dash curves) and 20 K (solid curves). (**g**) and (**h**) represent temperature dependence of the near surface XMCD peak height (negative) and XRMS peak height for samples S1 and S2, respectively.

XAS and XMCD measurements of the Mn *L* edge in an applied magnetic field of 700 Oe were taken at an angle of incidence of 10° from the plane of the film’s surface. Figure 4(a,b) show the Mn *L*_3_ and *L*_2_ edge total electron yield (TEY) average absorption (*I*^+^ + *I*^−^) spectra from S1 and S2, respectively, at 150 K (dash lines) and 20 K (solid lines). Similar profiles of XAS spectra have been observed at the intermediate temperature of measurements while warming and cooling the samples. Identical shapes of XAS spectra as a function of temperature suggest that the charge state and local electronic environment of Mn atoms at the surface of both the samples remains unchanged with temperature. However small change in XAS spectra near Mn *L*_3_ edge may be resulted from additional modification in surface electronic properties as suggested by off-specular XRR measurements. Figure 4(c,d) show the XMCD spectra from S1 and S2, respectively, at 150 K (dash lines) and 20 K (solid lines). The maximum of the (negative) XMCD signal at the Mn *L*_3_ edge can be seen at ~640.5 eV, which was the energy used for the off-specular XRMS experiments discussed later. Comparing XMCD data at lowest temperature measured (20 K), in the metallic region (Fig. 1) for both the samples, we observed the magnitude of the near-surface magnetization for the S1 is nearly 5% larger than that of the S2. Figure 4(e,f) show the XRMS spectra from S1 and S2, respectively, at 150 K (dash lines) and 20 K (solid lines). XRMS spectra from two samples at low temperature are quite different suggesting different magnetization depth profiles for the samples.

The total average magnetization in the surface region is proportional to the area bounded by the XMCD spectra^23^,^24^. We estimated the temperature dependent magnetization of the samples using XMCD and XRMS spectra. Figure 4(g,h) show the variation of normalized magnetization from XMCD and XRMS spectra from S1 and S2, respectively, while field cooling. The XMCD signal from S1 decreases faster than the XRMS signal from the same sample, with decreasing temperature. Whereas the opposite behavior was observed for S2. Thus the variations of near-surface magnetism of Mn with temperature depend on the chemical compositions and on the correlations of the phases of the surfaces.

**Figure 5:**
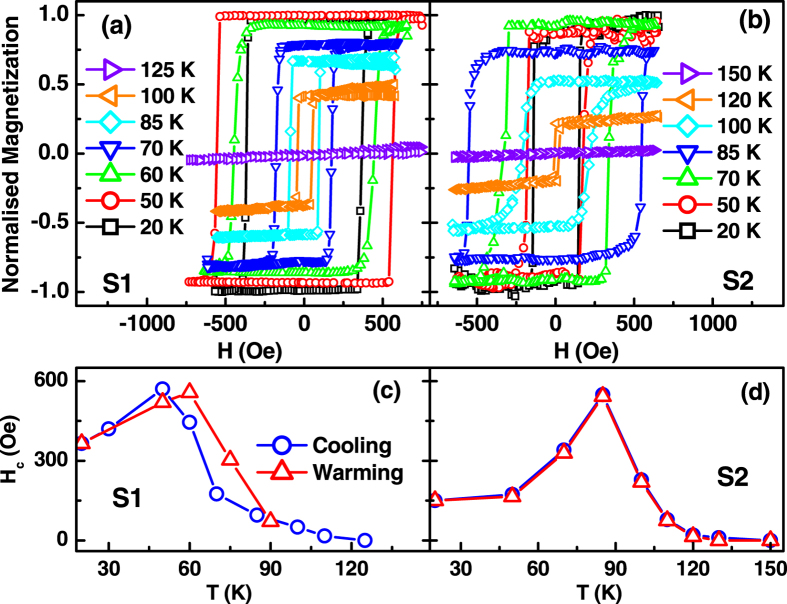
Normalized element selective hysteresis loops measured at the *L*3 edge of Mn (maximum XMCD, 640.5 eV) from S1 (**a**) and S2 (**b**) at different temperatures while cooling the samples. The angle of incidence is 10°. (**c**,**d**) show the variation of coercive field (*H*_c_) as a function of temperature for S1 and S2, respectively.

By measuring the specular reflectivity at maximum XMCD (E = 640.5 eV), hysteresis loops for the Mn moment can be obtained. Figure 5(a,b) depict the normalized element selective hysteresis loops measured at the *L*3 edge of Mn (maximum XMCD, 640.5 eV) from S1 and S2, respectively, at different temperatures while cooling the samples. We also measured these hysteresis curves while warming the samples. The temperature dependence of the coercive field (*H*_c_) of S1 and S2 are shown in the Fig. 5(c,d), respectively. We found thermal hysteresis of *H*_c_ about 11 K and ~0 K for S1 and S2, respectively, which is consistent with the thermal hysteresis of resistance (~18 K and 4 K) for S1 and S2.

### XRMS modeling and Off-specular X-ray resonant magnetic reflectivity

The XRMS, (*I*^+^ − *I*^−^), is the charge-magnetic interference term in the scattering amplitude and provides an alternative method for measuring the magnetic dichroism from the subsurface (within few ten of nm depth) region. In the soft x-ray regime, the longer wavelengths require calculation of the specular intensities using the magneto-optic boundary matrix formalism^38^,^39^. The charge-magnetic term in the scattering amplitude can be interpreted as interference between specularly reflected x rays from the chemical and magnetic structures.

**Figure 6:**
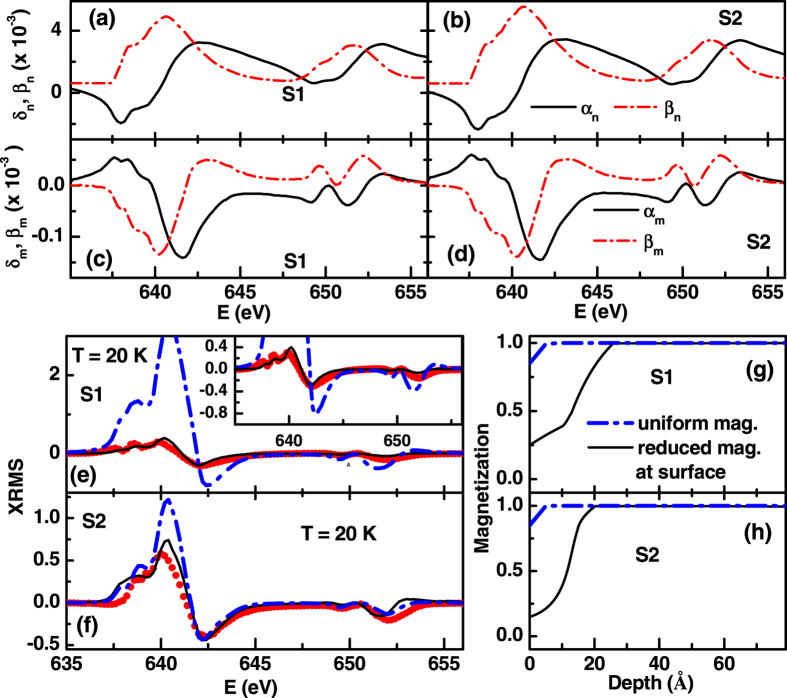
Optic constants as a function of energy for S1 (**a**) and S2 (**b**). Magneto-optic constants as a function of energy for S1 (**c**) and S2 (**d**). (**e**,**f**) show the specular XRMS data (solid circles) at an angle of incidence of 10° and corresponding fits (solid lines) for S1 and S2, respectively, at 20 K. (**g**,**h**) show the magnetization profiles (solid lines) for S1 and S2, respectively, which gave best fits (solid lines in (**e**,**f**)) to specular XRMS data. Dash-dot lines (blue) in (**g**,**h**) show the magnetization profiles with uniform magnetization which resulted in poor fits to the XRMS data (dash- dot blue lines in (**e**,**f**)).

Magnetization depth profiles were obtained from the energy-dependent XRMS using a magneto-optical matrix formalism developed by Zak *et al.*^38^ using the classical dielectric tensor. The formalism requires knowledge of the energy dependence of the refractive index, *n*^±^ = 1 − (*δ*_n_ ± *δ*_m_) + *i*(*β*_n_ ± *β*_m_) of the charge contributions, *δ*_n_ and *β*_n_, and the magnetic contributions, *δ*_m_ and *β*_m_. The optical (*δ*_n,_ *β*_n_) and magneto-optic (*δ*_m,_ *β*_m_) constants were obtained from XAS and XMCD measurement using the Kramers-Kronig transform^39^,^40^. Figure 6(a,b) show the optical constants (*δ*_n,_ *β*_n_) as a function of energy obtained from XAS spectra of S1 and S2, respectively at 20 K. Similarly Fig. 6(c,d) show the magneto-optic constants (*δ*_m,_ *β*_m_) as a function of energy as obtained from XAS spectra of S1 and S2, respectively at 20 K. We estimated optic constants and magneto-optic constants for S1 and S2 at different temperatures. Furthermore, we used the chemical structure, i.e. the thickness and roughness parameters, obtained from the non resonant XRR measurements, to analyze the XRMS data at different temperatures. Figure 6(e,f) show the XRMS data (closed circles) at 20 K and corresponding fit (solid line) from S1 and S2, respectively. The magnetization profile near the surface (few tens of nm) which best fit the XRMS data at 20 K for S1 and S2 are shown in Fig. 6(g,h), respectively. We obtained reduced magnetizations at the surfaces of both films—a result consistent with earlier PNR measurements on similar samples^20^,^21^. The reduced magnetization at the surface was attributed to presence of chemical non uniformity in this region^20^,^21^. For a comparison we have also fitted XRMS data (dash-dot blue lines in Fig. 6(e,f)) assuming uniform magnetization profile (dash-dot blue lines in Fig. 6(g,h)), which resulted in poor fit over whole energy range. Inset of Fig. 6(e) depicts the magnified version of Fig. 6(e), illustrating the difference between fittings of XRMS data at 20 K, assuming uniform and non uniform magnetization depth profile near surface.

**Figure 7:**
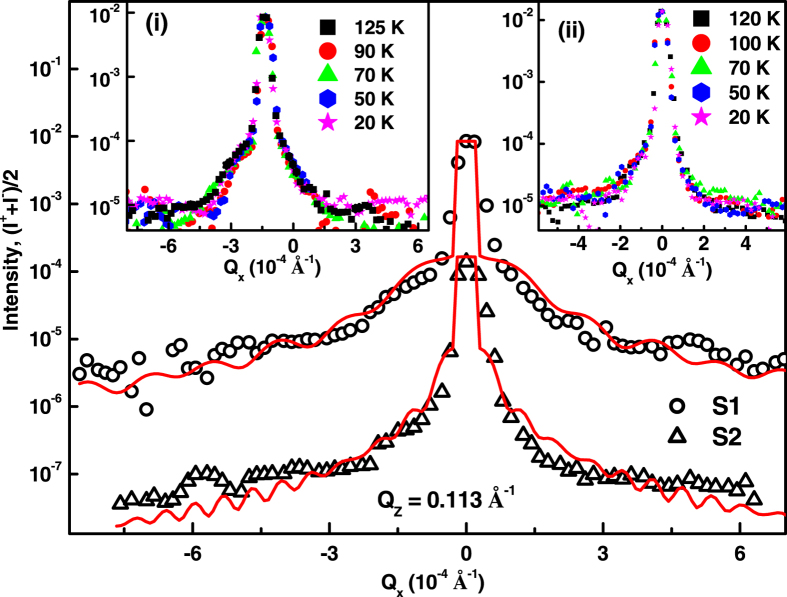
Diffuse charge scattering data, (I^+^ + I^−^)/2, as a function of Q_x_ from S1 (o) and S2 (Δ). Solid lines are fit to the data. The data have been shifted by 100. Inset (i) and (ii) show the diffuse charge scattering data, (I^+^ + I^−^)/2, at different temperatures from S1 and S2, respectively. The size of error bars on data is same as the size of data points.

Diffuse magnetic scattering can arise from both the fluctuation of the magnetic domains^41^ and spin^42^, which will manifest as magnetic roughness. Since the XRMS measurements were carried out at sufficiently high magnetic fields close to saturation the fluctuation of domain can be neglected and thus off-specular XRMS data can be treated within the same DWBA framework as for the laboratory based x-ray diffuse scattering measurements^43^,^44^. The mathematical descriptions of off-specular (diffuse) XRMS, specifically in reflectivity geometry have been discussed in detail elsewhere^45^,^46^,^47^,^48^,^49^. It has been demonstrated theoretically and experimentally that the diffuse difference (I^+^ − I^−^) intensity (charge-magnetic scattering) results predominantly from charge-magnetic cross-correlations while the diffuse sum (I^+^ + I^−^) intensity (charge scattering) results predominantly from charge-charge correlations^46^,^47^. The experimentally measured diffuse charge scattering data as a function of *Q*_x_, at fixed *Q*_z_ = 0.113 Å^−1^, at an energy of 640.7 eV, from S1 and S2, at different temperatures while warming is shown in the insets (i) and (ii) of Fig. 7, respectively. The diffuse charge scattering data at different temperatures remain similar suggesting that the charge-charge correlation length does not vary with temperature.

Using optic and magneto-optic constants at 640.7 eV estimated from the specular XRMS energy dependent data and other parameters (thickness, number density etc.) obtained from the hard XRR measurements, we fitted the diffuse (both charge and charge-magnetic) XRMS data at different temperatures with different morphological parameters (σ, *h* and ξ)^43^,^44^. The form of the correlation function for both the charge-charge and charge-magnetic fits is that for a self-affine fractal surface with a cutoff length^46^, i.e., 
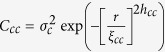
 and 
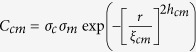
; where *ξ*_cc_ and *ξ*_cm_ are charge-charge and charge-magnetic correlation lengths and *h*_*cc*_ and *h*_*cm*_ are the corresponding Hurst parameters defining the texture of chemical and magnetic roughness^46^. Figure 7 shows the diffuse charge scattering data from S1 (open circles) and S2 (open triangle). The corresponding solid lines in Fig. 7 were fit to diffuse charge scattering data assuming similar morphological parameters (Table 1) as obtained from the non-resonant off-specular XRR data analysis (Fig. 3).

**Figure 8:**
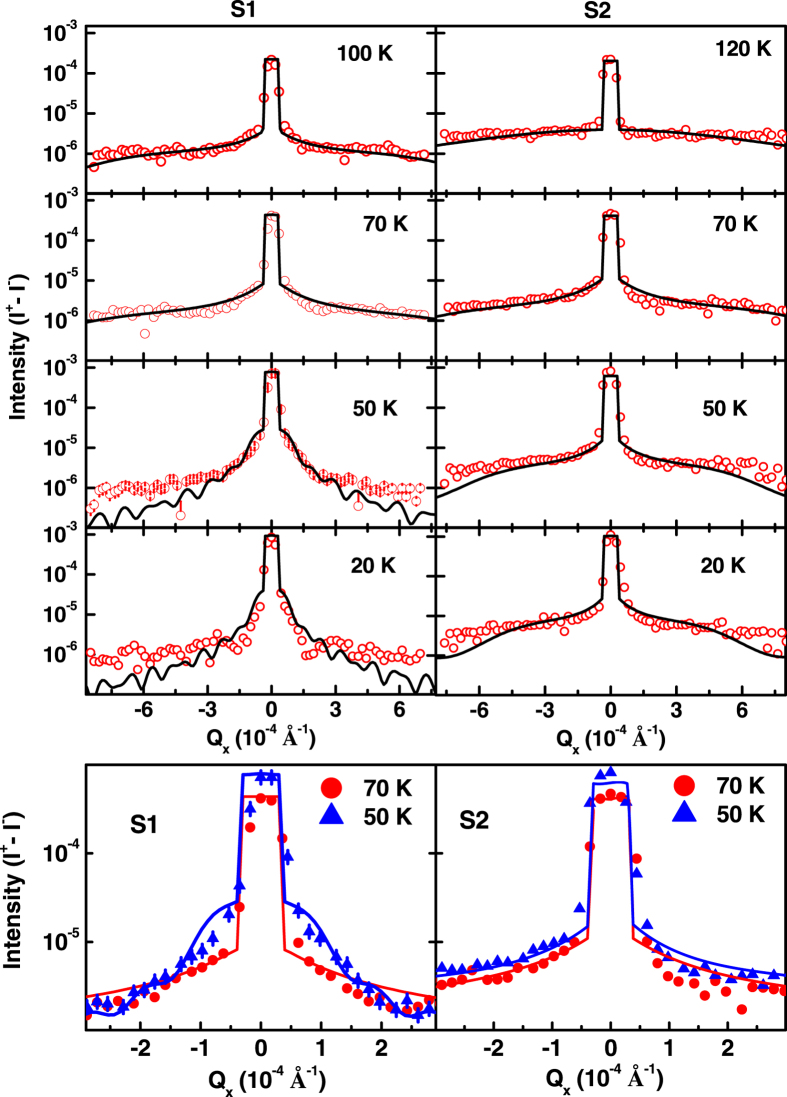
Diffuse charge-magnetic scattering data (open circles), (I^+^ − I^−^), and corresponding fits (solid lines) at different temperatures as a function of *Q*_x_ at fixed *Q*_z_ = 0.113 Å^−1^ from S1 (left panel) and S2 (right panel). Lower panels show the diffuse data and corresponding fit from S1 and S2 at 70 K and 50 K in lower *Q*_x_ range around specular ridge (*Q*_x_ = 0.0). The size of error bar on data is of same size of data points.

**Figure 9:**
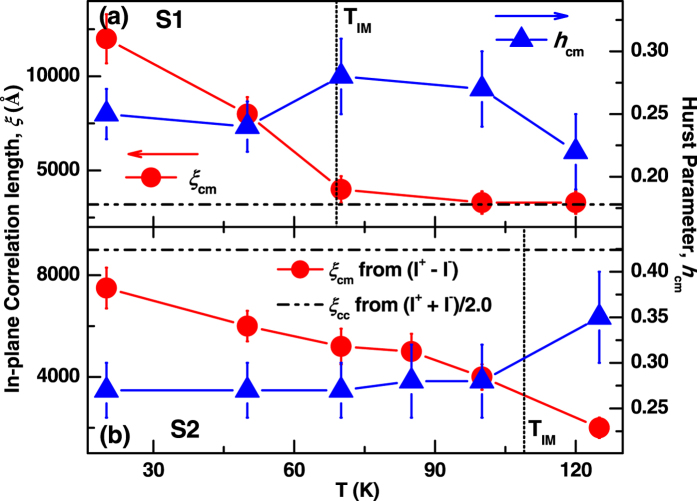
Morphological parameters (in-plane correlation length, ξ, and Hurst parameter, *h*) as a function of temperature obtained from diffuse charge-magnetic scattering data shown in Fig. 8 for S1 (**a**) and S2 (**b**).

Figure 8 shows the diffuse charge-magnetic scattering data as a function of *Q*_x_, at fixed *Q*_z_ = 0.113 Å^−1^, at a few temperatures while warming the samples S1 (left panel) and S2 (right panel) in a field of ~700 Oe. Solid lines in Fig. 8 are fits to the diffuse scattering data at different temperatures. We obtained smaller correlated root mean square roughness 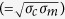 ~1.5 ± 0.6 Å as compared to *σ*_c,_ for charge-magnetic correlated surface which did not vary with temperature. Lower panels of Fig. 8 show a comparison of diffuse charge-magnetic scattering data (symbol) and corresponding fit (solid lines) at 70 K and 50 K, with in a smaller Q_x_ range near specular ridge, illustrating significant difference. The other morphological parameters (*h*_*cm*_ and *ξ*_cm_) obtained from these measurements are plotted as a function of temperature in Fig. 9(a,b) for S1 and S2, respectively. It is also evident from the Fig. 9 that the Hurst parameters obtained from diffuse charge-magnetic XRMS data remains invariant with temperature with average values of *h*_*cm*_ ≈ 0.25 ± 0.05 and 0.28 ± 0.05 for S1 [Fig. 9(a)] and S2 [Fig. 9(b)], respectively, - a result again similar to that obtained from charge scattering data for S1 (*h*_*cc*_ = 0.25 ± 0.03) and S2 (*h*_*cc*_ = 0.20 ± 0.03). The morphological parameters at few temperatures obtained from these measurements are shown in Table 2.

## Discussion

**Figure 10:**
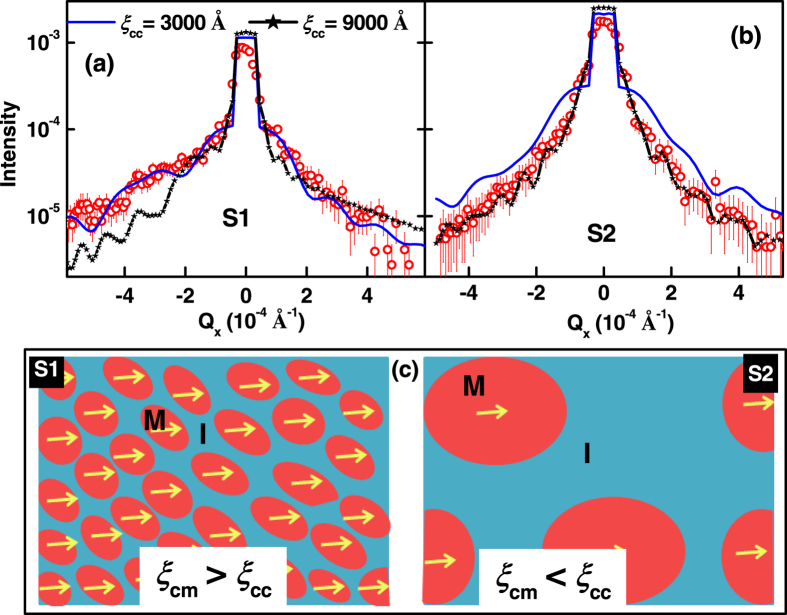
Off-specular XRR (non-resonant) from S1 (**a**) and S2 (**b**) and simulated profile with different correlation lengths as a function of Q_x_ at fixed Q_z_ = 0.17 Å^−1^. (**c**) shows the schematic of metallic and insulator phase map of S1 and S2 at low temperature.

The morphological parameters (in-plane correlation length and roughness exponent) obtained from non resonant Cu K_α_ off-specular XRR (Fig. 3) are purely due to charge scattering with negligible magnetic contribution. We obtained similar morphological parameters from non resonant Cu K_α_ off-specular XRR and soft x-ray diffuse sum (I^+^ + I^−^) intensity (charge scattering) measurements (Fig. 7). We obtained an in-plane charge-charge correlation length (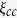) of ~3200 ± 400 Å and 9000 ± 800 Å for the LPCMO surface of S1 and S2, respectively, which is much larger as compared to that of the buried substrate-film interface (~500 ± 100 Å). A comparison of non resonant Cu K_α_ off-specular XRR data and corresponding fits assuming different 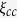 for S1 and S2 are depicted in Fig. 10(a,b), respectively, suggest different values of 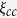 for the two samples. In addition both LPCMO surfaces showed a fractal surface with a Hurst parameter (roughness exponent) of ~0.22 (fractal dimension D ~2.7) for charge scattering data. Thus both S1 and S2 show similar roughness as well as a fractal surface (Table 1) but the charge-charge correlation length is drastically different, suggesting the slight composition change for LPCMO may lead to different long-range strain interactions and hence the correlations.

It is evident from Fig. 9 that the in-plane length scale, over which charge and magnetism are correlated, for S1 around 100 K–120 K (above MIT), is the same as the in-plane charge-charge correlation (~3200 ± 400 Å) length scale in the insulating phase. A rapid increase of the in-plane charge-magnetic correlation length 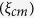 can be observed at temperature below 70 K (near *T*_MI_). We obtained higher 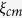 (~3–4 times of 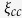) for S1 at temperatures below 70 K. Larger charge-magnetic correlation length 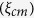 as compared to charge-charge correlation length 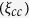 for a Fe/Gd multilayer using diffuse XRMS data was also observed earlier^46^. In contrast, we obtained smaller values of 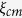 (~2000–7500 Å) than the 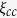 (~9000 ± 800 Å) for S2. However (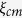) increases with decreasing temperature for both the samples. In addition similar Hurst parameters obtained for S1 and S2 from the diffuse charge-magnetic scattering data (Table 2), which are similar to the Hurst parameters obtained from charge-charge scattering data. *Thus, the distribution of magnetic moments possesses the same fractal dimension* (*D* ~*2.7*) *characteristic of the underlying chemical structure.*

Further, to know the correlation of magnetic domain length scales and the length scale over which chemical and magnetic roughness are correlated, we compared the results from cAFM measurements at 50 K and diffuse XRMS data at low temperatures (~50 K and 20 K) from S1 and S2. cAFM measurements at 50 K indicate different length scales for the two samples. S1 has smaller ferromagnetic (assuming the metallic phase is ferromagnetic) domains (~1700 Å) as compared to that of S2 (~12000 Å) at 50 K. 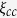 of S1 (~3200 Å) and S2 (~9000 Å) shows a similar trend and suggest that the surface roughness (or charge) are correlated to similar length scales. However, the 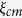 at 50 K for S2 (~6000 Å) is smaller than that of S1 (~8000 Å). The results suggest that charge and magnetic roughness are correlated to much higher (lower) length scale in S1 (S2) as compared to the metallic domain area observed by cAFM. Similar trends were observed when we compared the metallic phase obtained from cAFM and 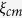 from diffuse XRMS at temperatures lower but near to *T*_MI_ for S1 (~50 K) and S2 (~85 K).

A typical metallic insulator phase map at low temperature as suggested by cAFM can be depicted in Fig. 10(c) for S1 and S2. Just below the MIT, the metallic phases in S1 are separated by smaller insulator regions as compared to S2, where we have larger metallic phase separated by large insulator regions. The different values of 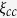 for S1 and S2, even though both the film showing similar *σ*_cc_ and *h*_cc_, may be due to doping, *x*, (different internal stress associated with doping) and different defects (stripes) etc., for different films. As temperature decreases below the MIT, the ferromagnetic (metallic) phases of S1 and S2 grow differently. In the case of S1, we have smaller ferromagnetic domains separated by smaller size non magnetic (insulator) phase (Fig. 10(c)) and hence the ferromagnetic domains may be correlated to larger length scale. Additionally the stripe domain in S1 may also enhance ferromagnetic correlation. Therefore the higher magnetic correlation length for S1 may also reflect in higher values of 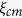 as compared to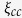. Whereas in the case of S2, the length scales of both metallic (ferromagnetic) and insulating phases are larger (Fig. 10(c)) as compared to that of S1 and hence show 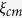 < 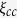.

It is noted that the composition variation for sample S1 and S2 is very small. However the internal stress associated with this small doping may influence the large long-range strain interactions in the films and hence resulting to different morphology and correlation length, since a strain dependent morphology for interfaces is well known in thin film. We observed different length scale over which charge and magnetism are correlated across MIT of S1 and S2. Different temperature dependent charge-magnetic correlation and related length scales shown by two samples may also influence the percolation of different phases across the MIT’s of these systems. Different percolation of conducting phase while heating and cooling cycle across MIT for LPCMO film grown identically as S1 was also observed earlier by cAFM^16^. Therefore different charge-magnetic correlation across MIT for these systems may be one reason for observing different thermal hysteresis behavior of macroscopic (transport, magnetism) properties.

## Conclusion

We measured the depth dependence of the chemical and magnetic structures as well as the in-plane charge-magnetic correlation length of the (La_0.4_Pr_0.6_)_1−x_Ca_x_MnO_3_ (LPCMO) films of similar thickness ~20 nm, with x = 0.33 (S1) and 0.375 (S2) across the MIT. We observed higher MIT for S2, which is ~40 K higher than that of S1. The large difference in MIT for S1 and S2 with similar thickness may be arising due to change in chemical pressure^11^ as a result of different compositions for two samples. We observed reduced surface magnetization for both the LPCMO films compared to the film bulk. The thermal hysteresis in resistance measurements (macroscopic) of the films is correlated with the thermal hysteresis of the coercivity as measured by specular XRMS from these manganite films. Using non-resonant (Cu K_α_) XRR data, we obtained in-plane charge –charge correlation length of 3200 Å and 9000 Å for S1 and S2, respectively, which were further confirmed by charge diffuse XRMS data. The temperature dependent charge diffuse XRMS data also confirmed that the in-plane charge-charge correlation lengths are independent of temperature. Using magnetic diffuse XRMS data we obtained an increase in the in-plane charge-magnetic correlation length (≥5000 Å) below the MIT’s for both samples S1 and S2. However, the charge-magnetic correlation length and charge-charge correlation length above the metal to insulator transition were the same for S1. In addition the in-plane charge-magnetic correlation length of S2 is always smaller than its in-plane charge-charge correlation length at all temperatures. Such a different correlation lengths for two films, which show similar roughness and roughness exponent, might have resulted due to different chemical pressure in the films for different doping. The variation of length scale over which the charge and magnetic phases are correlated may influence the percolation of different metallic/magnetic phases across metal insulator transitions of these systems and hence produce different thermal hysteresis of the transport and magnetic properties.

## Methods

### Sample Growth and characterization

Two single crystal (La_0.4_Pr_0.6_)_1−x_Ca_x_MnO_3_ (LPCMO) films with x = 0.33 and 0.375, hence forth known as samples S1 and S2, respectively, were epitaxially grown on (110) NdGaO_3_ (NGO) substrates in the step-flow-growth-mode using pulsed KrF laser (248 nm) deposition (PLD). During growth, the substrate temperature was 780 °C, O_2_ partial pressure was 130 mTorr, laser fluence was 0.5 J/cm^2^, and the repetition rate of the pulsed laser was 5 Hz^19^. Previously, scanning transmission electron energy-loss spectroscopy (EELS) microscopy^20^,^21^ found the composition of an identically prepared film (S1) to be (La_1−y_Pr_y_)_1−x_Ca_x_MnO_3_(y ~ 0.57, x ~ 0.27) averaged over the entire thickness of the sample. cAFM measurements were carried out on samples which were identically prepared as S1 and S2 and details of measurements are reported elsewhere^16^. The depth dependent structure and in-plane morphological parameters of the deposited films were obtained using non resonant (Cu K_α_ radiation, wavelength = 1.54 Å) specular and off-specular X-ray reflectometry, respectively.

### Resonant X-ray scattering

To study the electronic and magnetic properties of the LPCMO samples, two complementary soft x-ray techniques were used at beamline 4-ID-C of the Advanced Photon Source (APS): XMCD and XRMS^22^,^23^,^24^. The XMCD technique, measured through total electron yield (TEY), probes spin-dependent absorption. The photocurrents (scattering intensity) in XMCD (XRMS) measurements for right (RCP, *I*^+^) and left (LCP, *I*^−^) circular polarizations of the incident beam were measured independently. Both measurements were taken simultaneously by switching the polarization between LCP and RCP, across the Mn *L*_2,3_ edges at a fixed incident angle of 10°. Measurements were taken over a temperature range of 20–150 K, using in-plane fields of 700 Oe to saturate the magnetic moment of the sample. The sum (*I*^+^ + *I*^−^) (x-ray absorption spectroscopy, XAS) provides information on the electronic environment of the Mn 3d electrons. While magnetic information is contained in the difference (*I*^+^ − *I*^−^) signal, which in absorption and scattering are referred to as the XMCD and the XRMS, respectively^23^,^24^. We also measured the Off-specular (diffuse) XRMS from the samples at the Mn resonant energy of 640.5 eV.

## Additional Information

**How to cite this article**: Singh, S. *et al.* Composition dependence of charge and magnetic length scales in mixed valence manganite thin films. *Sci. Rep.* **6**, 29632; doi: 10.1038/srep29632 (2016).

## Supplementary Material

Supplementary Information

## Figures and Tables

**Table 1:** Parameters obtained from non-resonant specular and off-specular XRR.

System	Layer	Thickness (Å)	Correlated roughness (Å)	Correlation length, ξ, (Å)	Hurst parameter, *h*
S1	LPCMO	200 ± 10	3 ± 1	3200 ± 400	0.25 ± 0.03
	NGO Substrate	—	5 ± 1	500 ± 100	0.60 ± 0.10
S2	LPCMO	180 ± 10	4 ± 1	9000 ± 800	0.20 ± 0.03
	NGO Substrate	—	5 ± 1	500 ± 100	0.60 ± 0.10

**Table 2:** Parameters obtained from cAFM and diffuse XRMS at low temperature.

System	Temperature	cAFM	diffuse XRMS		
		Size of metallic (ferromagnetic) phase (Å)	In-plane Charge correlation length (Å)	In-plane charge-magnetic correlation length (Å)	Hurst Parameter (*h*)
S1	20 K	—	3200 ± 400	12000 ± 1100	0.25 ± 0.02
	50 K	1700	3200±400	8000 ± 850	0.24 ± 0.03
	85 K	1000	3200 ± 400	3500 ± 600	0.27 ± 0.03
S2	20 K	—	9000 ± 800	7500 ± 800	0.27 ± 0.03
	50 K	12000	9000 ± 800	6000 ± 650	0.27 ± 0.03
	85 K	9000	9000 ± 800	4900 ± 600	0.28 ± 0.03
