# Supplementary material for: Composition dependence of charge and magnetic length scales in mixed valence manganite thin films
Source: Sci Rep. 2016 Jul 27;6:29632. doi: 10.1038/srep29632 (PMC4995356; doi:10.1038/srep29632)
Supplement: Supplementary Information [file srep29632-s1.pdf]

Supplementary for Composition dependence of charge and magnetic length scales in mixed valence manganite thin films

Surendra Singh[1,*], J. W. Freeland[2], M.R. Fitzsimmons[3], H. Jeen[4,5] and A. Biswas[4]

[1]*Solid State Physics Division, Bhabha Atomic Research Center, Mumbai 400085 India*

[2]*Advanced Photon Source, Argonne National Laboratory, Argonne, Illinois 60439, USA*

[3]*Quantum Condensed Matter Division, Oak Ridge National Laboratory, Oak Ridge, TN, USA*

[4]*Department of Physics, University of Florida, Gainesville, FL 32611, USA*

[5]*Department of Physics, Pusan National University, Busan 609-735, Korea*

**surendra@barc.gov.in*

Two single crystal $(La_{0.4}Pr_{0.6})_{1-x}Ca_xMnO_3$ (LPCMO) films with x = 0.33 and 0.375, hence forth known as samples S1 and S2, respectively, were epitaxially grown on (110) $NdGaO_3$ (NGO) substrates using pulsed KrF laser (248 nm) deposition (PLD). Fig. S1 shows the transport (resistance), R (T), data from a number of samples with higher thickness (~ 270-350 Å) but equivalent composition as S1 (a) and S2 (b) grown on NGO substrates. Samples S1 and S2 show different thermal hysteresis of ~ 18 K and ~5K, respectively. However a small shift in metal to insulator transition (MIT) was observed for different samples with same compositions (see Fig. S1), which may be due to considerable variation in the thickness of the samples. Though small variation in other parameters e.g. oxygen variation etc. may affect the MIT. However EELS measurements on these samples suggested a uniform oxygen variation along the thickness of the samples and variation in O/Mn ratio was seen at interfaces of both thinner (~ 250 Å) and thicker (~ 350 Å) S1 films [S. Singh et al., *Phys. Rev. Lett*. 108, 077207 (2012).]. We found that MIT of S1 is lower by ~ 40 K then that of S2. Fig. S2 shows reduced magnetization [M(T)/M(10 K)] data measured using SQUID magnetometer as a function of temperature. Fig. S3 shows the transport measurements for similarly grown sample S1 (a) and S2 (b) on which cAFM measurements were carried out and discussed in manuscript. Inset shows the corresponding

AFM morphology of the surface of the films. Resistance, R(T), measurements are carried out using two probe resistance measurements. R(T) data shown in Fig. S1 and S3 are measured at LANL and university of Florida, respectively, and hence absolute value of resistance cannot be compared as the experimental errors e.g. distance between two probes, area of contacts etc., which influence the absolute value of the resistance, during two measurements were different. However, the MITs and associated thermal hysteresis, which are the intrinsic properties of the samples, show similar variations in Fig S1 and S3.

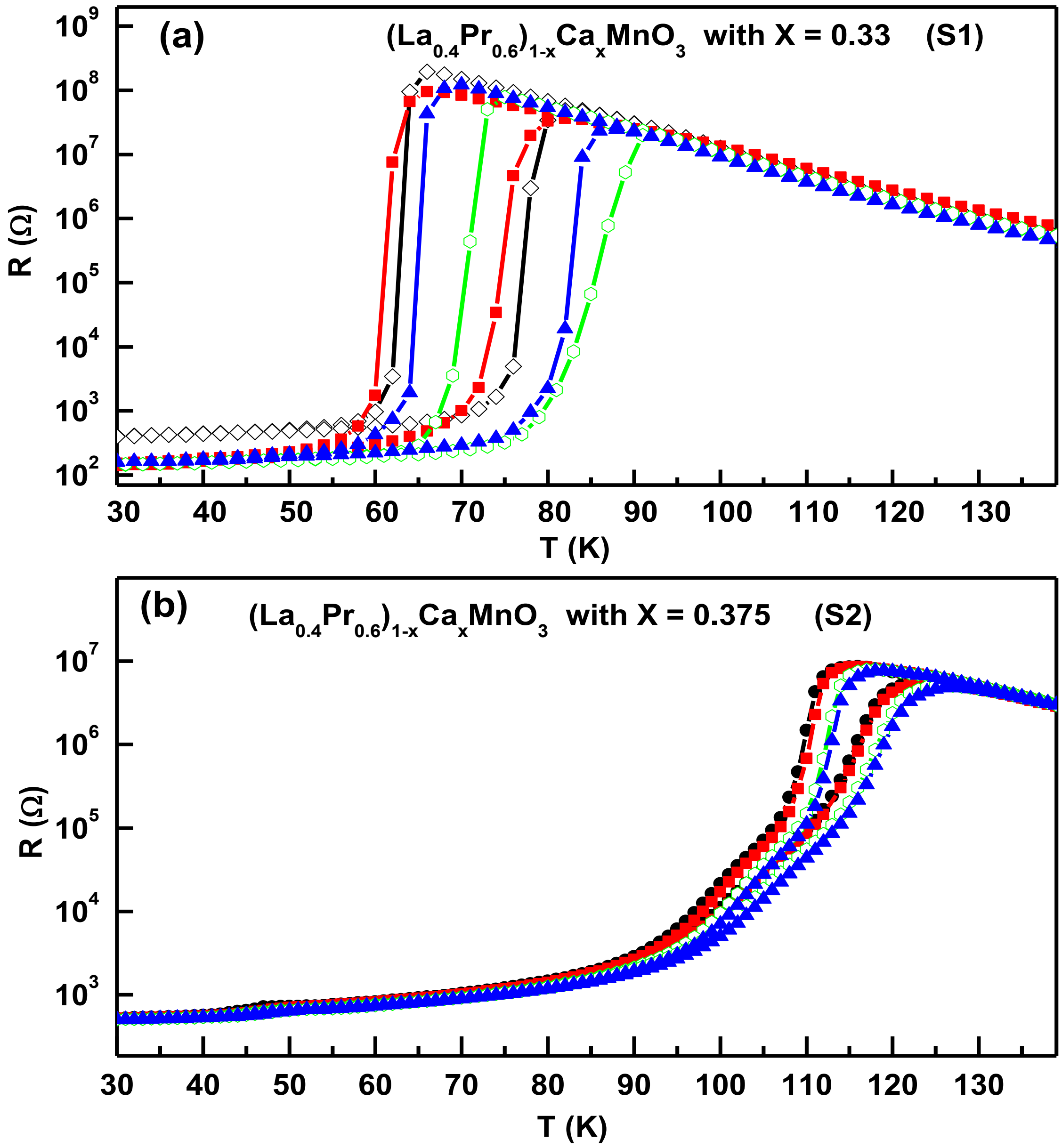

Fig. S1 : R(T) measurements from similarly grown samples S1 (a) and S2 (b).

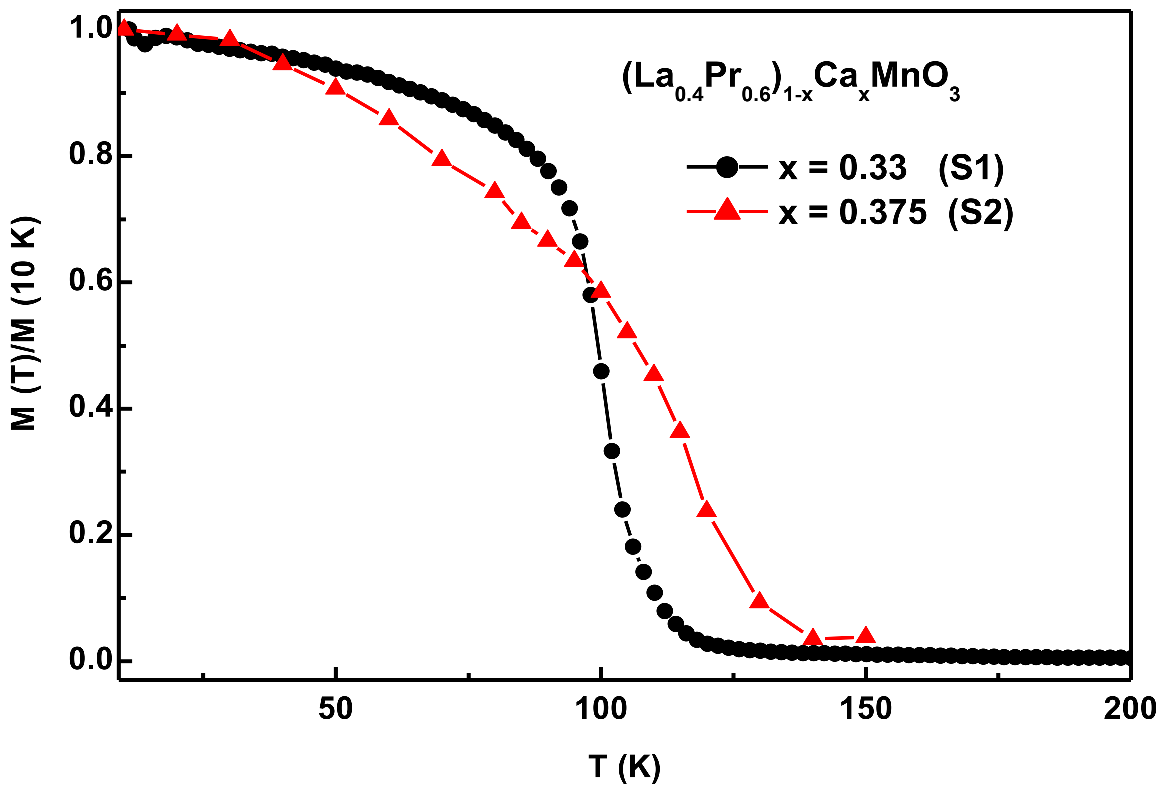

Fig. S2: Reduced magnetization as a function of temperature for similarly grown films S1 and S2.

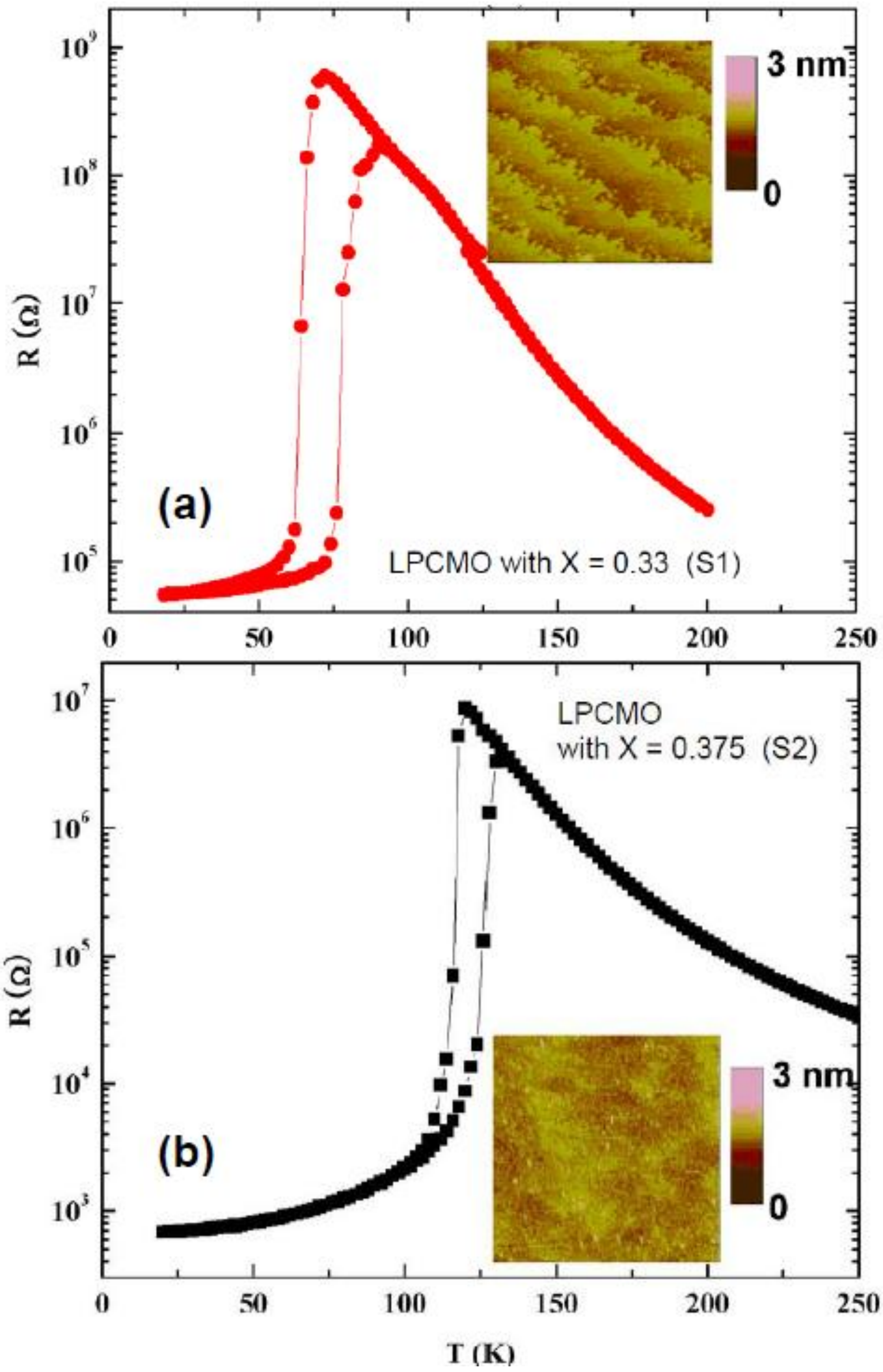

Fig. S3: transport measurements for similarly grown sample S1 (a) and S2 (b) on which cAFM measurements were carried out. Inset shows the corresponding AFM morphology of the surface of the films.
